# Determinants of Rural Household Resilience to Food Insecurity and Variations in Resilience Capacity across Livelihood Groups in Southwest Districts of Rural South Wollo, Ethiopia

**DOI:** 10.1016/j.cdnut.2026.109390

**Published:** 2026-06-06

**Authors:** Tadsual Asfaw Dessie, Mulugeta Amsalu Mekonnen

**Affiliations:** College of Agriculture and Natural Resources, Department of Rural Development and Agricultural Extension, Mekidela Amba University, Gimba, Ethiopia

**Keywords:** resilience capacity, adaptive capacity, access to basic services, household assets, social safety nets, Ethiopia

## Abstract

**Background:**

This study investigates the determinants of rural household resilience to food insecurity and examines variations in resilience capacity across socioeconomic and demographic groups in the southwest districts of South Wollo, Ethiopia.

**Objectives:**

This study examined and measured the determinants of rural household resilience to food insecurity and analyzed variations in resilience capacity across household characteristics and livelihood groups by applying the resilience index measurement and analysis II approaches.

**Methods:**

This study used a cross-sectional research design using primary data collected from 366 sample households. A 2-stage factor analysis model was applied. In the first stage, factor analysis was used to construct latent indices for the key resilience dimensions: access to basic services, adaptive capacity, assets, and social safety nets based on observed variables. In the second stage, a higher-order factor analysis was conducted to estimate an overall household resilience capacity index.

**Results:**

The results show that access to basic services and assets are the strongest contributors to household resilience, whereas social safety nets play the weakest role, reflecting limited coverage and unreliable support networks. Resilience levels differ significantly across districts, education categories, and household headship, with Legambo district, uneducated households, and female-headed households demonstrating the lowest resilience.

**Conclusions:**

The study concludes that resilience to food insecurity is shaped by structural constraints, including poor service access, restricted livelihood diversification, inadequate asset ownership, and weak social protection. The findings underscore the need for multidimensional, gender-responsive, and context-specific strategies to enhance rural household resilience in Ethiopia.

## Introduction

Food insecurity remains one of the most persistent development challenges globally, despite decades of policy attention and international commitment. Since its formal recognition at the World Food Conference in 1974, food security has continued to occupy a central place in global, national, and local development agendas, yet achieving it remains elusive, particularly in low-income and shock-prone regions. Recent reports indicate that hundreds of millions of people worldwide continue to experience hunger and undernourishment, with sub-Saharan Africa bearing a disproportionate share of the burden [1,2]. Structural poverty, climate variability, environmental degradation, and recurrent socioeconomic shocks have entrenched food insecurity across the region, resulting in both chronic and transitory forms of deprivation [[3], [4], [5], [6]].

Hereafter, Ethiopia exemplifies this challenge: with >80% of its population residing in rural areas and livelihoods heavily dependent on rain-fed agriculture, the country remains highly vulnerable to climatic shocks, seasonality, and long-term environmental stresses [7,8]. Recurrent droughts, land degradation, limited asset (AST) bases, and weak institutional support systems have contributed to persistent food insecurity and under nutrition, affecting millions of rural households [4,9]. Although policy efforts and social protection programs have yielded measurable progress over the past decades, food insecurity remains widespread, particularly in drought-prone areas. Particularly, the Amhara region, and South Wollo Zone in specifically, has consistently been identified as one of the most food-insecure areas of the country, characterized by recurrent climatic shocks, low agricultural productivity, high population pressure, and fragile livelihoods [[10], [11], [12]].

Although earlier research has primarily focused on assessing the status, severity, and determinants of food insecurity across various regions of Ethiopia, much of this literature remains limited to static measurements of food access and outcomes [[13], [14], [15], [16], [17], [18]]. They often fail to explain why some households are able to withstand and recover from shocks, whereas others fall deeper into food insecurity under similar conditions [19,20]. In response to this limitation, the concept of resilience to food insecurity has emerged as a promising analytical framework, emphasizing households’ capacities to anticipate, absorb, adapt to, and transform in the face of shocks and stresses [21,22].

In recent years, resilience-based approaches have gained considerable attention in food security and rural livelihood studies, particularly in Africa where recurrent climatic shocks, droughts, and economic instability continue to threaten household well-being [[23], [24], [25], [26], [27]]. Within this growing body of literature, the resilience index measurement and analysis (RIMA) framework developed by the FAO has become one of the most widely recognized and methodologically rigorous approaches for assessing household resilience to food insecurity [22]. The RIMA framework conceptualizes resilience as a multidimensional latent construct that reflects households’ capacities to withstand and recover from shocks through interconnected dimensions such as AST, adaptive capacity (AC), access to basic services (ABSs), and social safety nets (SSNs) [[28], [29], [30]].

Empirical studies conducted across African countries have successfully applied the RIMA approach to identify the key determinants of household resilience and evaluate the effectiveness of food security interventions [1,28,31,32]. Unlike conventional approaches that rely mainly on descriptive indicators or single-dimensional measures, RIMA uses a latent variable modeling approach that captures the complex and interrelated structure of resilience components [33,34]. Furthermore, studies applying the RIMA framework have shown that resilience is not a static outcome but a dynamic and context-specific process shaped by household resources, adaptive strategies, and institutional support systems [29,35].

The RIMA framework conceptualizes resilience as a multidimensional construct composed of several interrelated pillars, commonly including ABS, AST, AC, and SSN. By combining these components through a latent variable modeling approach, the framework allows researchers to quantify households’ capacity to withstand and recover from shocks while maintaining food security. Empirical applications of the RIMA framework across several African countries have demonstrated its effectiveness in identifying key drivers of resilience and informing evidence-based policy interventions aimed at strengthening food security in vulnerable rural contexts [1,28,31]. These studies highlight the importance of considering resilience as a dynamic and multidimensional process rather than a static outcome, thereby providing a stronger analytical basis for understanding long-term food security challenges.

Despite the growing prominence of resilience, empirical evidence on household-level resilience in Ethiopia remains fragmented. Existing studies often suffer from conceptual ambiguity, inconsistent terminology, and limited analytical rigor and fail to explicitly define resilience dimensions or rely on proxy indicators that do not adequately capture its multidimensional nature [36,37]. Methodologically, most studies use descriptive statistics or principal component analysis, which are insufficient for capturing the complex, interrelated, and hierarchical structure of resilience components [38,39]. Moreover, only a limited number of studies have applied internationally recognized frameworks such as the FAO’s RIMA model, which provides a standardized and theoretically grounded approach for measuring resilience at the household level [40,41]. Furthermore, empirical evidence from the southwest districts of South Wollo Zone remains limited. Because household resilience is highly context specific, shaped by local agroecological conditions, livelihood systems, AST endowments, and institutional settings, findings from other regions cannot be directly generalized to this area. This lack of localized and methodologically robust evidence constrains effective policy formulation and the design of targeted interventions to strengthen household resilience to food insecurity.

In response to this gap, the present study examines the determinants and distribution of rural household resilience to food insecurity in the southwest districts of South Wollo Zone, Ethiopia. Using the RIMA framework, the study provides a comprehensive, multidimensional, and standardized assessment of household resilience. By doing so, it addresses key geographical, conceptual, and methodological gaps in the existing literature. Specifically, the study identifies and measures the core dimensions and determinants of household resilience and analyzes variations in resilience capacity across household characteristics and livelihood groups. Accordingly, the study is guided by the following research questions:

(i) What are the key dimensions of rural household resilience to food insecurity in the study area?
(ii) How can household resilience to food insecurity be rigorously measured using the RIMA framework?

## Methods

### The study area

**FIGURE 1:**
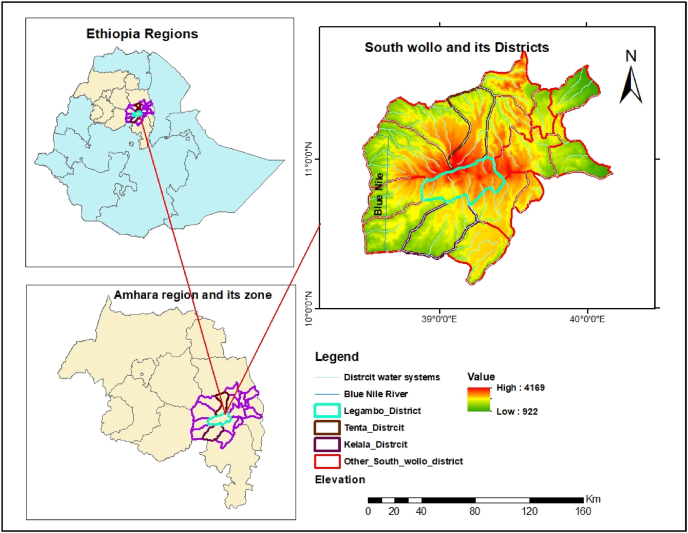
Location map of the study area. Source: Own extraction from Ethiopian map shape file, 2018.

The study was conducted in the southwest districts of the South Wollo Zone, Amhara National Regional State, Ethiopia, namely Legambo, Tenta, and Kelala, which were purposively selected from the 11 districts in the zone. The South Wollo Zone is geographically located between 10°10′ to 11°41′ N latitude and 38°28′ to 40°05′ E longitude. Agriculture, predominantly mixed crop–livestock farming systems, is the main source of livelihood across all 3 districts. Tenta district is located ∼529 km north of Addis Ababa, with altitudes ranging from 1100 to 1300 m above the sea level, mean annual rainfall of 500 to 950 mm, and mean temperatures between 17°C and 24°C. Legambo district lies ∼501 km north of Addis Ababa and is characterized by Wurchi (2.6%), Dega (48.4%), and Weina Dega (49%) agroecological zones, with altitudes ranging from 1500 to 3500 m above the sea level, annual rainfall of 700 to 1200 mm, and mean temperatures between 10°C and 20°C. Kelala district, also located in South Wollo Zone, has an altitudinal range of 500 to 2300 m above the sea level, receives a mean annual rainfall of ∼988 mm, and experiences temperatures ranging from 6°C to 29°C, with an average of 17°C. Figure 1 presents the geographical locations of the study districts within the South Wollo Zone of the Amhara Region, Ethiopia.

### Research design

The study applied a cross-sectional research design, collecting data from households at a single point in time. A household survey was used as the primary research strategy, with a focus on a quantitative approach. Specifically, the study used a sequential explanatory design, in which quantitative data collection and analysis were prioritized. The quantitative approach was applied to obtain information about rural households’ resilience to food insecurity using structured questionnaires.

### Sampling techniques and sample size determination

**FIGURE 2:**
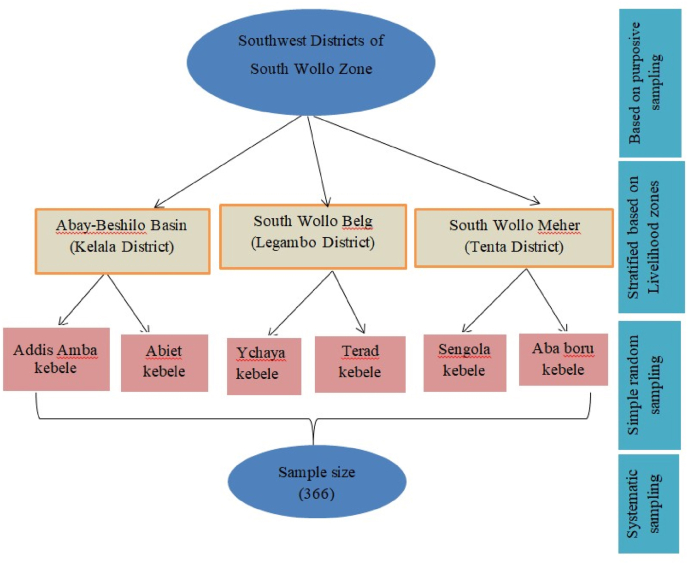
Multistage sampling procedure.

A multistage sampling technique was used to select the study area and sample households. The southwest districts of South Wollo Zone were purposively selected due to their chronic food insecurity status in the Amhara region, Ethiopia. The zone comprises 11 districts and 3 livelihood zones. First, the study area was stratified into 3 livelihood zones, Abay–Beshilo Basin, South Wollo Meher, and South Wollo Belg, based on the livelihood classification [42]. Second, 1 district was selected from each livelihood zone using stratified and proportional sampling. Third, 6 kebeles (kebele is the lowest administration system in Ethiopia) were selected from the chosen districts through proportional allocation and simple random sampling. Finally, households within each selected kebele were chosen using systematic random sampling with a fixed sampling interval (*k*), starting from a randomly selected household (Figure 2).

The sample size was determined using formula for finite populations described previously [43]. The 6 selected kebeles comprised a total of 7470 rural households. A 95% confidence level, ±5% precision, and a conservative population proportion (*p* = 0.5) were applied to ensure robustness. The formula provides a scientifically valid estimate while adjusting for finite population effects, as follows:

$$n=\frac{N.Z^{2}.p.q}{e^{2}\left(N-1\right)+Z^{2}.p.q}$$

where *n* = required sample size, *N* = total population size (7470 households), *Z* = Z-value at 95% confidence level (1.96), *p* = estimated proportion of the attribute in the population (0.5), *q* = 1 − *p*(0.5), and *e* = acceptable margin of error (0.05).

Thus, substituting the values into the formula gives the following:

$$n=\frac{7470{\left(1.96\right)}^{2}\left(0.5\right)\left(0.5\right)}{{\left(0.05\right)}^{2}\left(7470-1\right)+{\left(1.96\right)}^{2}\left(0.5\right)\left(0.5\right)}$$

$$n=\frac{7174.188}{19.6329}=365.6\approx 366$$

Thus, the final sample size required for this study is 366 rural households.

To maintain representativeness across kebeles, the total sample size (*n* = 366) was allocated proportionally based on each kebele’s household population size. This method ensures that larger kebeles receive a proportionally larger share of the sample and prevents sampling bias. The proportional allocation formula is follows:

$$n_{k}=\frac{N_{k}}{N}\times n$$

where: *n*_*k*_ = sample size for kebele *k*, *N*_*k*_ = total households in kebele *k*, *N* = 7470 (total households), and *n* = 366 (required sample size). The resulting kebele-level distribution is presented in Table 1.

**TABLE 1:** Sample size for the selected kebeles

District	Kebele	Total households (*N*_*k*_)	Sample (*n*_*k*_)
Legambo	Ychaya	1800	88
	Terad	1020	51
Kelala	Addis Amba	1200	59
	Abiet	1150	56
Tenta	Aba Boru	1250	61
	Segola	1050	51
Total	6 kebeles	7470	366

### Methods of data sources and collection

This study primarily relied on primary data collected from sampled rural households in the study area. Because the analysis focused on household-level food insecurity, the household head was considered the unit of analysis. Primary data were collected through structured interview schedules administered to household heads or responsible adult members. In addition, secondary data were obtained from the agricultural offices of the Legambo, Tenta, and Kelala districts.

### Method of data analysis

#### Identifying dimensions household resilience using RIMA measurement

The first and to date most widely used quantitative measure of resilience is RIMA. The method was substantially updated and re-released as RIMA-II [22]. As describes in detail further, FAO (RIMA-II) uses the 4 latent variables, ABS, AST, SSN, and AC from household survey data. Each of these dimensions of resilience by themselves too is latent variables estimated using observable household-level variables. Table 2 presents the observed variables for each dimension of RIMA measures of resilience capacity that are used in resilience analysis with their expected relationships. Table 2 presents the observed variables used for each RIMA resilience dimension and their expected relationships with resilience capacity. The ABS dimension includes household access to credit, health, market, and extension services, as well as distance to the nearest district town measured in kilometers. AC is represented by 7 observed variables: diversification of household income sources, diversification of livestock owned by the household, diversification of crops cultivated on household agricultural land, and the literacy status of the household head. The AST dimension is constructed using 4 indicators: per capita agricultural land size measured in hectares, livestock ownership measured in tropical livestock units, agricultural AST index, and nonagricultural AST index. Furthermore, SSN, the final latent dimension of resilience, is represented by 2 observed variables: formal and informal cash transfers received by households during the previous 12 months. Together, these indicators capture the multidimensional nature of household resilience capacity.

**TABLE 2:** Explanatory variables of RIMA dimensions and their expected effects

Resiliencecapacitydimensions	Variable description	Expectedeffecton resilience
ABS	Access to credit services (yes = 1)	+
	Access to health services (yes = 1)	+
	Access to market services (yes = 1)	+
	Access to extension services (yes = 1)	+
	Distance to nearest district town in (KM)	−
AC	Income diversification index	+
	Livestock diversification index	+
	Crop diversification index	+
	Ability to read and write (1 = yes)	+
	Agricultural asset index	+
AST	Nonagricultural asset index	+
	Size of agricultural land measuredin hectare	+
	Livestock number in TLU per capita	+
SSN	Cash transfers from formal source in ETB	+
	Cash transfers from informal sources in ETB	+

*Source:* FAO, 2016. +, positive relationship; −, negative relationship. Abbreviations: ABS, access to basic service; AC, adaptive capacity; AST, asset; RIMA, resilience index measurement and analysis; SSN, social safety net; TLU, tropical livestock unit; KM, Is Killo Meter; ETB, Ethiopian Birr.

#### Measuring household resilience using RIMA approach

To estimate household resilience capacity, the study used the RIMA framework using a 2-step factor analysis (FA) procedure. As noted by Bollen [44], latent variable approaches assume that ≥1 unobserved variable generate the relationships observed among measurable indicators. In this study, resilience capacity is considered a latent construct that cannot be directly observed but can be inferred from a set of proxy indicators.

In the first stage, exploratory factor analysis (EFA) was conducted to estimate the latent dimensions of household resilience capacity from observed indicators. EFA was used because the underlying structure among the indicators was not imposed a priori but was identified empirically from the data. This procedure allowed the reduction of multiple proxy indicators into a smaller number of latent dimensions representing key components of resilience capacity.

To achieve a simpler and more interpretable factor structure, a varimax orthogonal rotation was applied. The rotation helped maximize the loading of each variable on a single factor while minimizing crossloadings across factors, thereby improving the interpretability of the extracted dimensions. The resulting factors correspond to the major resilience pillars commonly used in the RIMA framework. A formal expression of this idea is as follows:

(1) $$\Upsilon_{i}=\lambda_{0}+\lambda_{1}\varepsilon_{i,1}+\lambda_{2}\varepsilon_{i,2}+\ldots +\lambda_{k}\varepsilon_{i,k}+\mu_{i}$$

where;${\;\Upsilon }_{i}$**=** observed indicator for the *i*th case; $\lambda_{0}$**=** intercept term; **λ_1_…λ_k_** = factor loadings (coefficients) associated with the *k* latent variables; $\varepsilon_{i,1}\ldots \varepsilon_{i,k}$ **=** values of the first through *k* latent variables for case *i*; and $\mu_{i}$ = unique variable or error term. Therefore, pillars such as ABS, SSN, AC, and AST were constructed based on this notion.

Based on the study objectives, key substantive variables influencing household resilience were identified and used to measure resilience capacity. Prior to analysis, the suitability of the data for FA was assessed using established diagnostic tests, including the Kaiser–Meyer–Olkin (KMO) measure of sampling adequacy, Bartlett test of sphericity, checks for multicollinearity or singularity, and evaluation of factor loadings [[45], [46], [47], [48], [49]].

The KMO test confirmed sample adequacy, as values >0.5 indicate suitability for FA, as described by Kaiser [49] cited in Gambo Boukary et al. [50]. Bartlett test of sphericity rejected the null hypothesis that the correlation matrix is an identity matrix, indicating sufficient correlations among variables for FA [48,51]. Multicollinearity and singularity were assessed using the determinant of the correlation matrix, which exceeded the recommended threshold of 0.00001, suggesting the absence of serious multicollinearity, as suggested by Field [47], cited in Atara et al. [38]. Moreover, factor retention followed Kaiser’s eigenvalue-greater-than-1 criterion to identify meaningful factors, as described by Kaiser [49] cited in Kebede et al. [52]. Regarding factor loadings, their interpretation depends on sample size, with higher loadings required for smaller samples [47,53]. Given the sample size of 384, factor loadings >0.364 were considered acceptable, confirming the adequacy and robustness of the FA results [47].

In the second stage, a second-order FA was performed using the estimated latent dimensions to construct the household resilience capacity index (RCI). In this step, the 4 resilience pillars, ABS, ASTs, AC, and SSNs, were combined to generate a composite resilience index. This hierarchical approach allows the individual dimensions to contribute jointly to the overall household resilience capacity. The combined scores of RCI can be expressed as follows:

(2) $${RCI}_{i,t}=f\left({ABS}_{i,t}{{AC}_{i,t}AST}_{i,t}{SSN}_{i,t}\right)+\varepsilon_{i,t}$$

where RCI of *i*th household depends on the level of ABS, AC, AST, and SSN at times.

As mentioned earlier, this study used FA stated in terms of equation, and in FA context, the abovementioned functional relationship takes the form as follows:

(3) $${\text{RCI}}_{i}=f\left({W_{\text{ABS}}\;\text{ABS}}_{i}{{+W_{\text{AC}}\text{AC}}_{i}+W_{\text{ASS}}\text{AST}}_{i}{+W_{\text{SSN}}\text{SSN}}_{i}\right)$$

where ${\text{RCI}}_{i}$ = resilience of the household, ${\;\text{ABS}}_{i}$ = access to basic services, ${\text{AC}}_{i}$= adaptive capacity, ${\text{AST}}_{i}$= asset, and ${\text{SSN}}_{i}$= social safety nets.

Although loadings are expressed as weights, they are rarely used directly to compute component or resilience scores in practice [47]. Instead, 2 adjustments are made as follows: the variables are standardized, and the factor loadings are converted into factor score coefficients (also known as regression coefficients). These coefficients are then applied to the standardized variables to estimate component scores. Accordingly, Equation *3* was transformed into Equation *4* as follows, which was used to estimate the resilience index in this study.

(4) $$CI=\sum wc\left(\frac{x_{\dot{i}}-x_{j}}{s_{i}}\right)$$

where ${RCI}_{i}$= estimated resilience index for household *i*, wc = the weight (factor score coefficient) for the FA, $x_{i}$= the *i*th of the household values for the *x*th variable (indicators); note: the *x*th in the current study’s context are the variables denoted by ${ABS}_{i\;},{{AC}_{i\;},ASS}_{i},{\text{and}\;SSN}_{i\;}$ in Equation *1*; $x_{j}$= the mean of *x*th variable for overall households and $s_{i}$ = SD of the *x*th variable for overall households. To make these data organized for analysis, all quantitative datasets was coded and entered into SPSS, version 25 (due to its quality for data management; IBM Corp) and exported into STATA version 14.1 (due to its quality for executing and using statistical models; STATA Corp).

## Results and Discussion

### Measuring rural household resilience to food insecurity

To achieve this objective, the study applied FAO’s RIMA framework, a widely used and validated approach for assessing household resilience to food insecurity. RIMA conceptualizes resilience as a multidimensional and latent construct, measured indirectly through observable household-level indicators. Accordingly, a latent variable approach was used to combine multiple indicators into broader resilience dimensions relevant to the study area. The analysis identified 4 core resilience dimensions consistent with the RIMA framework: ABS, AST, SSN, and AC. Each dimension was estimated from multiple indicators, capturing different aspects of household resilience. A 2-stage FA was then used: first, factor loadings were extracted for each resilience dimension, and second, these dimensions were aggregated into a single RCI. This index provides a quantitative measure of households’ ability to withstand, absorb, and recover from food insecurity shocks.

### Access to basic services

ABS is a key pillar of rural household resilience to food insecurity under the RIMA framework, reflecting households’ connectivity to essential financial, market, health, and institutional services. Consistent with resilience literature [21,22], better access to services such as credit, markets, health care, and extension enhances coping capacity, livelihood stability, and adaptive potential. In this study, ABS was assessed using 6 indicators: access to credit and savings institutions, market, health and extension services, and distance to the nearest district town.

EFA was conducted to identify the underlying structure among these indicators. The number of factors retained was determined primarily based on the inspection of the scree plot and the interpretability of the rotated factor loadings, aiming to achieve a clear and meaningful factor structure. Although eigenvalues are reported in Table 3, they were used only as supporting information rather than as the sole decision rule for factor retention. Based on the screen plot and the coherence of the factor loadings after rotation, 2 factors were retained, which together explained ∼69.6% of the total variance.

**TABLE 3:** Factor extraction results (eigenvalues) of access to basic services

Factor	Eigenvalue	Difference	Proportion	Cumulative
Factor 1	2.61849	1.06175	0.4364	0.4364
Factor 2	1.55675	0.64979	0.2595	0.6959
Factor 3	0.90695	0.42305	0.1512	0.8470
Factor 4	0.48390	0.21476	0.0806	0.9277
Factor 5	0.26914	0.10437	0.0449	0.9725
Factor 6	0.16477	—	0.0275	1.0000

Likelihood ratio test—independent vs. saturated: χ^2^(15) = 920.29; probability > χ^2^ = 0.0000.

Before factor extraction, all statistical requirements for a reliable FA model were examined and satisfied (Table 4). The KMO value of 0.582 exceeded the minimum acceptable threshold, whereas Bartlett test of sphericity was highly significant (χ^2^ = 917.76; *P* < 0.001), confirming adequate intercorrelations among variables. In addition, the determinant value (0.079) suggested no serious multicollinearity concerns, indicating the suitability of the dataset for FA. These findings are consistent with previous resilience studies [31].

**TABLE 4:** Factor loadings of access to basic services

Variables	Factor 1	Factor 2	Uniqueness
Access to credit services	0.8606	−0.1217	0.2445
Availability of saving institutions	0.6770	0.4817	0.3096
Access to market services	−0.5664	0.7211	0.1593
Access to extension services	0.2571	0.3881	0.7833
Access to health services	0.6208	0.6818	0.1497
Distance to nearest district town in kilometers	0.8045	−0.4176	0.1783

Bartlett test of sphericity: χ^2^ = 917.760; df = (15); *P* = 0.000. Kaiser–Meyer–Olkin measure of sampling adequacy = 0.582. Determinant of *R*-matrix = 0.079. *Source:* Author’s computation using household survey data (2025).

As presented in Table 4, the rotated factor structure reveals important patterns and underlying social dynamics that extend beyond the descriptive statistics. The first factor loads strongly on access to credit services, availability of savings institutions, access to health services, and proximity to district towns, representing a broader dimension of formal institutional and financial accessibility. The clustering of these variables suggests that households located closer to district centers are not only geographically advantaged but are also embedded within stronger institutional networks that facilitate access to multiple services simultaneously. This pattern indicates that spatial location may shape resilience through cumulative service advantages rather than through isolated interventions.

The second factor is characterized primarily by strong loadings for market access and health services, suggesting a dimension related to market participation and service utilization. However, the loading pattern also reveals a notable contrast. Although market access demonstrates a strong association with this factor, extension services show weak loadings and high uniqueness (0.7833), indicating limited integration with the broader service system. This finding suggests that extension services may function independently or unevenly across households and may not effectively connect households with financial, market, and institutional support structures. Such fragmentation raises concerns about institutional coordination and may indicate that service delivery systems are operating in parallel rather than as integrated mechanisms for strengthening resilience.

Another noteworthy finding is the crossloading of health services across both factors. Unlike other indicators, health access contributes simultaneously to institutional accessibility and market-service engagement, implying that health care availability intersects with multiple dimensions of household well-being. This dual association reflects the central role of health in resilience formation, since healthier households are better positioned to participate in markets, engage in productive activities, and utilize available opportunities. These findings are consistent with previous studies in Ethiopia and similar contexts [39,54], which emphasize that resilience emerges through interconnected systems of support rather than through isolated service provision.

### AC

AC reflects households’ ability to adjust to changing environmental and socioeconomic conditions and represents a key dimension of resilience within the RIMA framework. It captures the capacity of households to modify livelihood strategies, diversify income sources, and use human capital to respond to shocks and long-term changes. In this study, AC was measured using 4 observed indicators: income diversification index, crop diversification index, livestock diversification index, and the literacy status of the household head. These indicators capture both human capital and livelihood flexibility. The income diversification index reflects households’ engagement in multiple income-generating activities such as crop and livestock production, trade, remittances, and income from perennial and vegetable crops. Crop diversification was measured based on the dominant crops cultivated in the study districts, whereas livestock diversification was constructed from the major livestock species kept by households. Together with literacy status, these indicators were used to estimate the latent construct of AC using EFA.

The number of factors retained was determined primarily through inspection of the scree plot and the interpretability of the rotated factor loadings, with the objective of obtaining a clear and meaningful factor structure. Based on these criteria, 2 factors were retained, jointly explaining ∼63.11% of the total variance in the AC indicators (Table 5). Eigenvalues for each factor are presented in Table 5 as descriptive information.

**TABLE 5:** Factor extraction results (eigenvalues and variance explained) of adaptive capacity

Factor	Eigenvalue	Difference	Proportion	Cumulative
Factor 1	1.39460	0.26482	0.3486	0.3486
Factor 2	1.12978	0.24388	0.2824	0.6311
Factor 3	0.88589	0.29616	0.2215	0.8526
Factor 4	0.58974	—	0.1474	1.0000

Likelihood ratio test—independent vs. saturated: χ^2^(6) = 70.03; probability > χ^2^ = 0.0000.

Model diagnostics further support the suitability of the FA (Table 6). Bartlett test of sphericity was statistically significant (χ^2^ = 69.835; df = 6; *P* < 0.001), indicating that the correlation matrix was appropriate for factor extraction. The KMO measure of sampling adequacy was 0.470, which is slightly below the commonly recommended threshold of 0.50, indicating that the shared variance among the included variables is relatively modest. This relatively low KMO value may be attributed to the heterogeneous nature of AC indicators, which capture diverse socioeconomic, institutional, and livelihood characteristics that are not always strongly correlated in rural household settings. In addition, the Bartlett test of sphericity remained statistically significant, indicating that the correlation matrix was appropriate for FA despite the relatively low KMO value. Therefore, the dimension was considered acceptable for inclusion in the resilience analysis, although the result should be interpreted with caution.

**TABLE 6:** Factor loadings of adaptive capacity

Variables	Factor 1	Factor 2	Uniqueness
Income diversification index	0.0445	0.7743	0.3985
Crop diversification index	0.8243	0.0993	0.3107
Livestock diversification index	–0.0001	0.7355	0.4590
Literacy rate	0.8307	–0.0499	0.3075

Bartlett test of sphericity: χ^2^ = 69.835; df = (6); *P* = 0.000. Kaiser–Meyer–Olkin measure of sampling adequacy = 0.470; determinant of *R*-matrix = 0.823.

Moreover, as presented (Table 6), the rotated factor structure uncovers important relationships and contrasts that extend beyond the descriptive statistics. Factor 1 loads strongly on crop diversification and literacy status, representing a dimension associated with human capital and productive knowledge systems. The close association between literacy and crop diversification suggests that education may enhance households’ ability to adopt new agricultural practices, process information, and make strategic production decisions. Literate households may possess greater access to information and a stronger capacity to respond to changing climatic and market conditions through diversified crop choices. Factor 2 is characterized by strong loadings for income diversification and livestock diversification, reflecting a broader livelihood diversification dimension. The clustering of these variables indicates that households often rely on multiple economic activities and livestock ASTs as complementary risk management strategies. Livestock diversification may provide a buffer against crop failures, whereas income diversification reduces dependence on a single livelihood source.

Moreover, the findings demonstrate that AC is inherently multidimensional and reflects the interaction of knowledge resources, livelihood diversity, and household decision-making processes. Strengthening household resilience therefore requires integrated interventions that simultaneously promote education, support diversified livelihood opportunities, and address structural constraints that limit households’ ability to transform available resources into adaptive outcomes. These findings align with previous studies showing that human capital and livelihood diversification jointly enhance resilience to climatic and economic shocks [37,[55], [56], [57]].

### AST index

The AST index was analyzed as a latent variable representing household resilience, capturing both income- and non–income-related ASTs that support livelihoods. It was constructed from the following 4 observed variables: per capita agricultural land, livestock holdings in tropical livestock units, agricultural AST index, and nonagricultural AST index.

EFA was conducted to identify the underlying structure among AST-related indicators. The number of retained factors was determined through scree plot inspection and the interpretability of rotated factor loadings. Based on these criteria, 1 factor was retained, explaining ∼50.11% of the total variance among AST indicators (Table 7). This result suggests that household ASTs largely converge around a common resilience dimension while still retaining some degree of heterogeneity in how individual AST types contribute to food security. Model diagnostics confirm the suitability of the factor model (Table 8). The likelihood ratio test comparing the independent and saturated models was statistically significant (χ^2^ = 221.24; df = 6; *P* < 0.001), indicating that the variables share sufficient correlations to justify factor extraction.

**TABLE 7:** Factor analysis results for asset index variables

Factor	Eigenvalue	Difference	Proportion	Cumulative
Factor 1	2.00438	1.16578	0.5011	0.5011
Factor 2	0.83859	0.14860	0.2096	0.7107
Factor 3	0.69000	0.22297	0.1725	0.8832
Factor 4	0.46703	—	0.1168	1.0000

Likelihood ratio test (independent vs. saturated model): χ^2^(6) = 221.24; *P* = 0.000.

**TABLE 8:** Factor loadings of asset index variables

Variable	Factor 1 loading	Uniqueness
Agricultural asset index	−0.5495	0.6980
Nonagricultural asset index	0.7925	0.3719
Livestock (TLU per capita)	0.7746	0.4001
Agricultural land size (hectares)	0.6888	0.5256

Abbreviation: TLU, tropical livestock unit.

The factor loading structure reveals important patterns that extend beyond simple AST ownership. Nonagricultural ASTs and livestock ownership exhibit the strongest positive associations with the latent AST dimension, whereas agricultural land size also contributes substantially. This pattern suggests that resilience is not determined solely by traditional agricultural resources but increasingly depends on diversified forms of wealth accumulation. Livestock and nonfarm ASTs may provide households with more flexible and liquid resources that can be mobilized during periods of crisis. Unlike fixed land resources, these ASTs can be converted into cash or used strategically to smooth consumption and absorb shocks.

More importantly, the results reveal a notable contradiction in the role of agricultural ASTs. Although ownership of farming tools and agricultural equipment is generally expected to strengthen resilience, the agricultural AST index demonstrates a negative loading on the primary AST factor. This finding suggests that agricultural tools and equipment do not align with the broader AST accumulation pathway represented by livestock and nonfarm wealth. One possible explanation is that households possessing greater agricultural equipment may remain highly dependent on farming activities and, therefore, remain more exposed to climate variability and production-related shocks. In contrast, households with stronger livestock and nonagricultural AST bases may possess broader livelihood opportunities and greater flexibility to withstand shocks. These findings are consistent with previous studies conducted in Sidama, Damot Pulasa, and the northeastern Ethiopian highlands, which emphasize that productive AST ownership is a key mechanism for buffering shocks and strengthening resilience [38,39,58,59].

### Social safety nets

SSNs represent another key dimension of household resilience, capturing the support households receive from formal institutions and informal social networks during times of shock or hardship. Within the RIMA framework, SSNs reflect the extent to which households can rely on social protection programs and community-based support mechanisms. In this study, SSNs were measured using 2 indicators: cash transfers from formal sources and cash transfers from informal sources. Formal transfers primarily include government or institutional programs, whereas informal transfers capture assistance from relatives, friends, and community networks.

Given the limited number of variables, EFA identified 1 underlying dimension representing household access to social support. The eigenvalues and variance proportions are presented in Table 9 for descriptive purposes. The retained factor explains ∼52.15% of the total variance.

**TABLE 9:** Eigenvalue and proportions of social safety nets

Factor	Eigenvalue	Difference	Proportion	Cumulative
Factor 1	1.04309	0.08618	0.5215	0.5215
Factor 2	0.95691	—	0.4785	1.0000

Likelihood ratio test—independent vs. saturated: χ^2^(1) = 0.13; *P* = 0.7155.

Model diagnostics indicate that the determinant of the correlation matrix (0.998) suggests no multicollinearity issues among variables (Table 10). The KMO value (0.500) met the minimum acceptable threshold, although the Bartlett test was not statistically significant (*P* = 0.717), which likely reflects the small number of variables included in this dimension.

**TABLE 10:** Correlations of social safety nets with variables

Variables	Corrective SSN index
SSN index	1.0000
Cash transfers from formal sources	0.4761
Cash transfers from informal sources	0.9087

Bartlett test of sphericity: χ^2^ = 24.624; df = 1; *P* = 0.717. Kaiser–Meyer–Olkin measure of sampling adequacy = 0.500; Determinant of the *R*-matrix = 0.998. *Source:* Author’s computation using household survey data (2025).

Abbreviation: SSN, social safety net.

Moreover, as shown in Table 10, correlation analysis indicates that both formal (*r* = 0.476) and informal transfers (*r* = 0.909) positively contribute to household resilience, with informal transfers playing a particularly significant role. The correlation structure reveals an important contrast between formal and informal support mechanisms. Both formal and informal transfers positively contribute to household resilience, but informal transfers display a substantially stronger relationship with the SSN dimension. This finding suggests that community-based support systems and social relationships play a more influential role in household coping strategies than institutional programs.

Furthermore, the findings indicate that household resilience in rural areas depends not only on formal social protection systems but also on the strength of community-based social capital. Strengthening resilience therefore requires greater integration between institutional safety net programs and local social support structures to create more inclusive and responsive support systems. These findings are consistent with previous studies, emphasizing the importance of remittances and community-based support mechanisms in strengthening household resilience [28,40,60].

### Aggregating household resilience capacity to food insecurity

After estimating each resilience dimension separately, a second-stage FA was conducted to compute the overall household RCI. The latent variables from the first-stage analysis, ABSs, AC, ASTs, and SSNs, served as input. Statistical tests confirmed the suitability of the data: Bartlett test was significant (χ^2^ = 151.858; df = 6; *P* < 0.001), KMO was 0.569, and the determinant of the correlation matrix was 0.114, indicating no multicollinearity.

Using the Kaiser criterion, 1 factor was retained with an eigenvalue of >1, explaining 62.42% of the total variance (Table 11). Factor loadings (Table 12) show that ABSs (0.9615) and ASTs (0.8054) are the strongest contributors to resilience, highlighting the importance of service access, land, livestock, and equipment. AC has a high negative loading (−0.8637), reflecting factor orientation rather than effect direction, confirming the importance of income, crop, livestock diversification, and human capital. SSNs have a weaker contribution (0.4217), indicating that transfers provide short-term support but are less central to long-term resilience.

**TABLE 11:** Eigenvalues and variance explained for the overall resilience capacity index

Factor	Eigenvalue	Difference	Proportion	Cumulative
Factor 1	2.49682	1.57401	0.6242	0.6242
Factor 2	0.92281	0.44587	0.2307	0.8549
Factor 3	0.47694	0.37352	0.1192	0.9741
Factor 4	0.10343	—	0.0259	1.0000

Likelihood ratio test (independent vs. saturated model): χ^2^(6) = 154.03; *P* = 0.0000.

**TABLE 12:** Factor loadings of overall resilience capacity index

Variable	Factor 1	Uniqueness
Access to basic services	0.9615	0.0756
Adaptive capacity index	−0.8637	0.2541
Assets index	0.8054	0.3514
Social safety nets	0.4217	0.8221

Bartlett test of sphericity: χ^2^ = 151.858; df = 6; *P* = 0.000. Kaiser–Meyer–Olkin measure of sampling adequacy = 0.569. Determinant of the correlation matrix = 0.114. *Source:* Author’s computation using household survey data (2025).

Furthermore, factor loadings show that ABS is the strongest contributor to overall resilience (0.9615). The particularly strong influence of ABSs reveals an important systemic characteristic of rural resilience. Household capacities do not operate independently of broader institutional environments. Households with better access to markets, credit facilities, and social services are likely to benefit simultaneously from multiple forms of support, creating cumulative advantages that strengthen resilience over time. This finding suggests that geographical and institutional inequalities may generate uneven resilience outcomes across households. Thus, resilience may depend not only on household resources but also on the surrounding service infrastructure within which households operate. These findings are consistent with evidence from Ethiopia [54,61,62].

The AST index also has a strong positive loading (0.8054). The AST dimension also exhibits a strong positive contribution to resilience, reinforcing the role of land, livestock, and productive resources in enabling households to absorb and recover from shocks. However, the earlier analysis of AST composition revealed that not all ASTs contributes equally. Livestock and nonagricultural ASTs showed stronger associations than agricultural tools, suggesting that resilience depends not only on AST ownership itself but also on the flexibility and convertibility of those ASTs. Therefore, the importance of ASTs in the aggregate model reflects both wealth accumulation and households’ capacity to mobilize resources during periods of crisis. These results are aligned with the previous studies of [40,63,64].

A notable finding emerges from the AC dimension, which demonstrates a high negative loading (−0.8637). Statistically, the negative sign reflects factor orientation rather than indicating a detrimental effect on resilience. Nevertheless, the magnitude of this loading reveals the substantial importance of AC within the overall resilience structure. More importantly, the opposite directional loading may indicate an underlying contrast between adaptive strategies and structural resource endowments. Although access to services and ASTs represents relatively stable and accumulated forms of resilience, AC reflects dynamic behavioral responses such as diversification and human capital utilization. The results are consistent with previous findings [56,59,65].

SSNs exhibit the weakest loading (0.4217), indicating a comparatively limited contribution to the shared resilience structure. This result suggests that transfers and social assistance programs play a more peripheral role in shaping long-term resilience. Although safety nets may provide critical support during periods of hardship, their relatively weaker association implies that they primarily function as short-term coping mechanisms rather than as drivers of sustained resilience.

**FIGURE 3:**
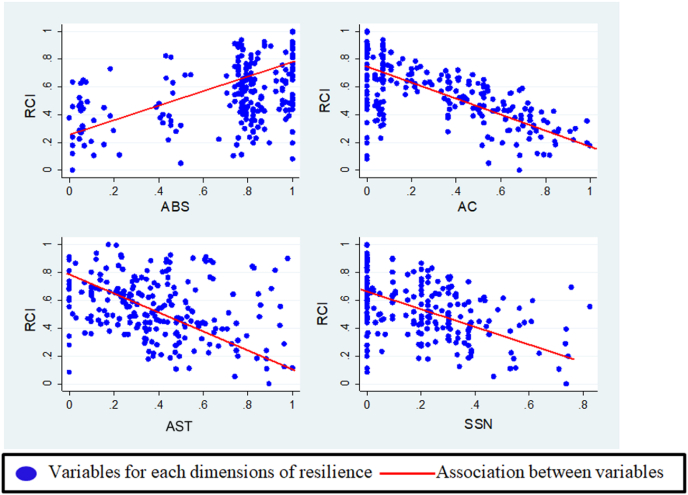
Distribution of overall resilience capacity index (RCI) using scatter plots. ABS, access to basic service; AC, adaptive capacity; AST, asset; SSN, social safety net.

A 2-way scatter plot was used to examine the relationships between the 4 RIMA dimensions—ABS, AC, AST index, and SSN—and the overall RCI (Figure 3). All indices were standardized using minimum–maximum normalization for comparability. The plots show a strong, near-linear positive relationship between ABS and the RCI, indicating that better access to services is a key driver of household resilience, consistent with the FA and previous studies in Ethiopia [54,61,62]. AC also shows a positive but more dispersed relationship with resilience, reflecting heterogeneity in livelihood diversification and human capital across households [40,56,59]. The AST index exhibits a clear positive association with the RCI, highlighting the buffering role of land, livestock, and other ASTs in coping with shocks [63,65]. In contrast, SSNs show a weaker relationship with resilience, suggesting that while transfers support short-term coping, they contribute less to long-term structural resilience [40,41].

### Profile of household resilience capacity

After constructing the overall household RCI through second-stage FA, this study examined variations in resilience across key household characteristics. Profiling resilience helps identify structural differences among population groups and the main sources of vulnerability and strength. Accordingly, resilience capacity was compared across districts, gender of the household head, and educational level. Table 13 presents the mean household resilience capacity of sample households across the 3 study districts.

**TABLE 13:** Means of overall household resilience capacity

Variables	Mean	SD
Districts		
Legambo	0.0872	0.0498
Kelala	0.7932	0.2031
Tenta	0.5961	0.1957
Sex of the households		
Male	0.4752	0.3925
Female	0.3336	0.3266
Education status		
Uneducated	0.3944	0.3576
Educated	0.3000	0.3139

### Overall resilience capacity across study districts of sample household

**FIGURE 4:**
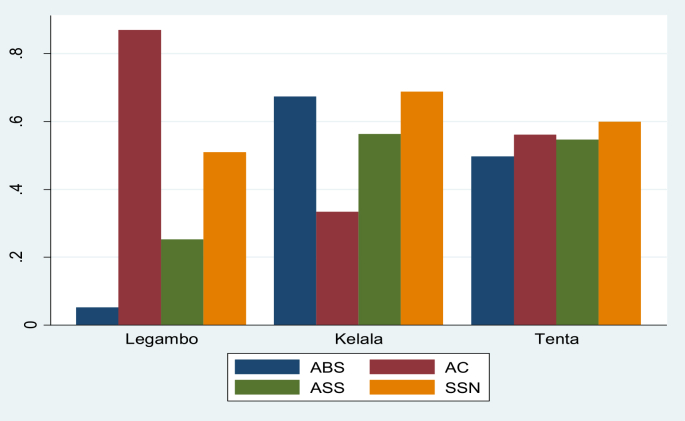
Resilience pillar distribution by districts. ABS, access to basic service; AC, adaptive capacity; AST, asset; SSN, social safety net.

Figure 4 compares household resilience across Legambo, Kelala, and Tenta using the 4 RIMA dimensions: ABS, AC, ASTs, and SSN. Clear spatial disparities are evident. Kelala consistently shows the highest resilience across all dimensions, particularly in ABS, reflecting better access to credit, markets, health, extension, and savings services, which strengthens coping capacity [41,66]. Tenta exhibits moderate resilience, whereas Legambo records the lowest scores, indicating substantial service and livelihood constraints. AC and AST ownership follow a similar pattern, with Kelala outperforming the other districts due to greater livelihood diversification, literacy, and AST accumulation, whereas Legambo remains the most vulnerable [56,59,63,64]. SSNs show weaker variation but still favor Kelala. Overall, the results highlight the need for localized and context-specific interventions, particularly in structurally disadvantaged districts such as Legambo [40,59].

### Overall resilience capacity by educational status of the household head

**FIGURE 5:**
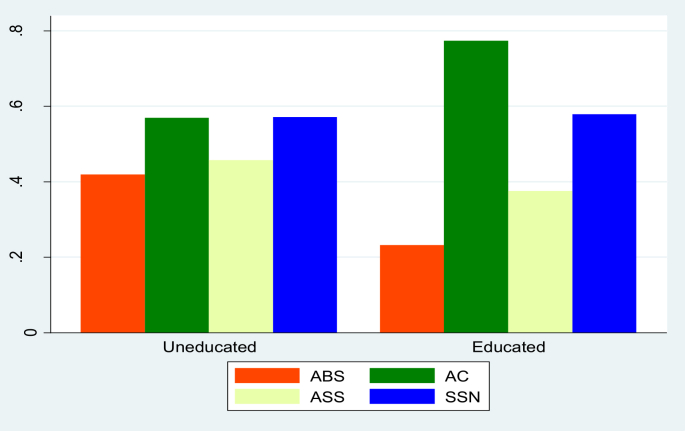
Resilience pillar distribution by household education status. ABS, access to basic service; AC, adaptive capacity; AST, asset; SSN, social safety net.

Figure 5 shows clear differences in household resilience between educated and uneducated household heads across all RIMA dimensions. Education emerges as a strong determinant of resilience, with the largest gap observed in ABS, where educated households have significantly higher scores due to better awareness, decision making, and engagement with institutions [61]. Educated households also exhibit stronger AST ownership, reflecting improved income opportunities and long-term accumulation of land, livestock, and productive ASTs [38,55]. Similarly, AC is higher among educated households, indicating greater livelihood diversification, information access, and adoption of improved practices. Differences in SSNs are smaller, suggesting broad access to informal support; however, educated households retain a slight advantage. Overall, the results highlight education as a foundational pillar of household resilience and emphasize the importance of investing in rural education to strengthen food security [41].

### Overall resilience capacity by sex of the household head

**FIGURE 6:**
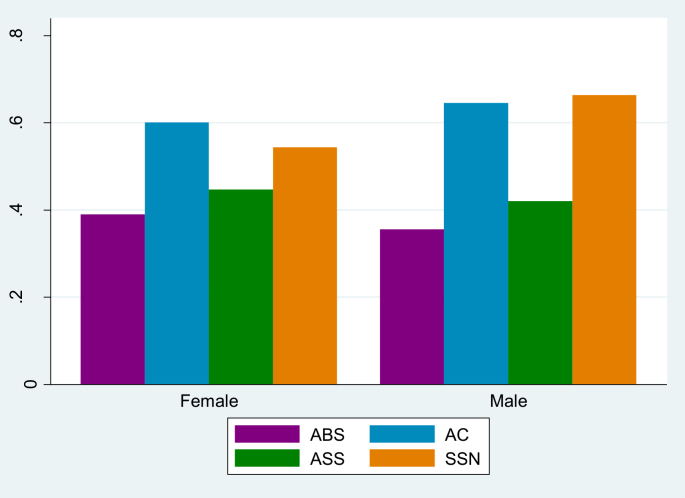
Resilience pillar distribution by sex of the household. ABS, access to basic service; AC, adaptive capacity; AST, asset; SSN, social safety net.

Figure 6 reveals clear gender disparities in household resilience, with male-headed households outperforming female-headed households across all RIMA dimensions. The largest gap appears in the AST dimension, where male-headed households hold more land, livestock, tools, and savings, reflecting structural constraints on women’s access to ASTs, credit, and inheritance [40,65]. Male-headed households also show better ABSs and higher AC, due to fewer institutional barriers and greater access to information, training, and livelihood diversification opportunities [56,63]. Differences in SSNs are smaller, indicating the importance of informal support for female-headed households, although males often benefit from stronger formal networks [60].

## Conclusion

This study assessed rural household resilience to food insecurity in the southwest districts of South Wollo Zone using FAO’s RIMA-II framework. The results show that the 4 resilience pillars contribute unevenly to overall resilience. ABSs and AST ownership were the strongest determinants of households’ ability to withstand food insecurity shocks, whereas SSNs had the weakest effect due to limited coverage and inconsistency. AC was moderately strong, driven by literacy and livelihood diversification but constrained by low education levels and limited income opportunities. Clear disparities in resilience were observed across districts and household characteristics. Kelala exhibited the highest resilience, followed by Tenta, whereas Legambo remained the most vulnerable. Households headed by educated and male household heads demonstrated significantly higher resilience than those headed by uneducated and female heads, reflecting persistent structural inequalities in access to resources and services. Hence, the findings underscore the need for targeted, context specific, and gender-responsive interventions to effectively strengthen household resilience to food insecurity.

### Recommendation

Based on the findings, the following key recommendations are proposed to enhance household resilience and food security:

✓ Improve ABSs by strengthening rural infrastructure and institutions, including credit, markets, roads, and health facilities, with priority to Legambo district.
✓ Promote livelihood diversification through climate-smart agriculture, small-scale irrigation, nonfarm activities, and expanded extension services to build AC.
✓ Support AST accumulation for vulnerable households, particularly female-headed and low-AST households, by improving access to productive ASTs and technologies.
✓ Strengthen SSNs by scaling up the Productive Safety Net Program (PSNP), ensuring timely transfers, and reinforcing informal systems such as idir and ikub.
✓ Invest in education and literacy as long-term resilience strategies by expanding adult education, improving school attendance, and integrating nutrition and resilience education.
✓ Adopt district-specific interventions reflecting local vulnerability patterns, with targeted investments in Legambo and AC building in Tenta.
✓ Enhance research and monitoring through continuous resilience assessment and longitudinal studies to track changes and evaluate intervention impacts.

## Author contributions

The authors’ responsibilities were as follows—TAD and MAM: designed the research; TAD and MAM: conducted the research; TAD: analyzed the data; TAD and MAM: wrote the manuscript; TAD: had primary responsibility for the final content; and all authors: read and approved the final version of the manuscript.

## Declaration of generative AI and AI-assisted technologies in the writing process

The author(s) declare that no generative AI or AI-assisted technologies were used in the writing of this manuscript.

## Data availability

Data described in the manuscript and code book will be made publicly available through the NIH data repository National Heart, Lung, and Blood Institute BioData Catalyst (BDC). Analytic code will be made available on request.

## Funding

The authors reported no funding received for this study.

## Conflict of interest

The authors report no conflicts of interest.
